# Conceptualisation and Development of a values-based scale of emergency physicians’ professional identities

**DOI:** 10.1186/s12909-023-04376-0

**Published:** 2023-06-02

**Authors:** Yu-Che Chang, Nothando Sithulile Nkambule, Xaviera Xiao, Lynn Valerie Monrouxe, Hsu-Min Tseng

**Affiliations:** 1 Chang Gung Medical Education Research Centre (CGMERC), Chang Gung Memorial Hospital, Linkou, Taoyuan, Taiwan; 2 Department of Emergency Medicine, Chang Gung Memorial Hospital, Linkou, Taoyuan, Taiwan; 3 Chang Gung University College of Medicine, Taoyuan, Taiwan; 4 International Graduate Program of Education and Human Development, National Sun Yat-sen University, Yat-sen, Taiwan; 5 Clinical Competency Center, Chang Gung University of Science and Technology, Taoyuan, Taiwan; 6 The Faculty of Medicine and Health, School of Health Sciences, University of Sydney, Sydney, NSW Australia; 7 Department of Health Care Management, Chang Gung University, Taoyuan, Taiwan

**Keywords:** Emergency Physicians, Professional identities, Scale development, Values

## Abstract

**Background:**

Physicians’ values about what constitute their professional identities are integral in understanding how they ascribe meaning to their practice. However, there is no general consensus on the conceptualization and measurement of physicians’ professional identities. This study developed and validated a values-based scale for measuring physicians’ professional identities.

**Methods:**

A hybrid research method was used to gather both qualitative and quantitative data. We employed literature review, semi-structured interview, Q-sorting exercise to examine the conceptualization of emergency physicians’ professional identities and to initially develop a 40-item scale. A panel of five experts assessed the scale’s content validity. Using 150 emergency physicians as our sample, we conducted Confirmatory Factor Analyses (CFA) to test the fit of our hypothesised four-factor model based on our preliminary findings.

**Results:**

Initial CFA suggested revisions to the model. Following theoretical assumptions and modification indices, the model was revised and adjusted to a four-factor 20 item Emergency Physicians Professional Identities Value Scale (EPPIVS) with acceptable fit statistics χ2 = 389.38, df = 164, Normed χ2 = 2.374, GFI = 0.788, CFI = 0.862, RMSEA = 0.096. The Cronbach’s alpha, McDonald’s Omega reliability and composite reliability of the subscales ranged from α: 0.748 to 0.868, Omega: 0.759 to 0.868 and CR: 0.748 to 0.851, respectively.

**Conclusion:**

The results indicate that the EPPIVS is a valid and reliable scale for measuring physicians’ professional identities. Further research on the sensitivity of this instrument to important changes over career progression in emergency medicine is warranted.

**Supplementary Information:**

The online version contains supplementary material available at 10.1186/s12909-023-04376-0.

## Background

Professional identities help individuals assign meaning to professional practices, boost morale and influence role enactment [1, 2]. This has significant implications in an era of prevalent global health crises. Poor identification with one’s profession is associated with poor job satisfaction, high burnout rates and increased medical errors; endangering patient safety [3]. Consequently, curricular reforms that teach the practice of medicine as well as the act of being a physician are encouraged [1, 4, 5]. Assessing the efficacy of such reforms requires rigorous measures. However, ambiguity in the conceptualization of professional identities hinders the development of rigorous and context-based measures [6–8].

Without a general consensus on what comprises a valid and conceptually robust measure of professional identities, the problem of how to reliably measure physicians’ professional identities will persist [6, 9, 10]. Moreover, without reliable measures, empirical evidence on how diverse backgrounds influence physicians’ level of identification with their profession will remain impalpable [5, 11]. Therefore, scholars call for more reliable, practical, context sensitive conceptualizations of professional identities along with objective indicators to be used to underscore the measurement of the construct [6, 12].

A central issue in the quantitative assessment of professional identities for physicians lies in ascertaining what underpins professional identities, the different domains that make up the construct and in developing a measure that will reflect a unified theoretical perspective of these domains. A growing body of literature indicates that, professional values are essential for group identification [2] particularly in uncertain times or when roles change and blur as this can affect which, when and how professional values are enacted [13]. As such, the hierarchy we place on our values determines what we perceive to be important, helping us set priorities and rationalise our decisions, actions and behaviours [13, 14]. In turn this facilitates professionals’ abilities to resolve conflicts that arise when making decisions about best practices in different context [15, 16]. Yet to date, within healthcare professions, only the nurse value scale [17] and nurse match [18] are values-based measures of professional identities.

The professional development and retention of emergency physicians remains a central issue, challenging the development of emergency medicine as a specialty [19–21]. Given the growing concern over the sustainability of emergency physicians’ careers [20], this study aims to develop a values-based professional identities scale tailored for emergency physicians. In terms of research, this scale will provide a valuable measure to be utilised with other measurement tools (e.g. examining stress, burnout) to ascertain potential vulnerabilities and strengths across members of this workforce [e.g. 22]. Finally, insights from using this scale may be a catalyst for devising measures to attract and retain emergency physicians [19, 20]. Therefore, we set out to address the following research questions:

RQ1: What values underpin emergency physicians’ professional identities and how do the different conceptualisations of these values reflect the underlying latent variables of the construct?

RQ2: How viable, reliable, and valid are our hypothesised latent variables of emergency physicians’ professional identities?

## Methods

### Study design and setting

Our study is a multi-method cross-sectional study outlining the process of developing and evaluating the psychometric properties of a scale designed to measure emergency physicians’ professional identities. The scale assesses the values held by emergency physicians regarding emergency care practice. This study is part of a project aimed at understanding physicians’ professional identities, burnout and resilience based on a sample of emergency physicians from Chang Gung Memorial Hospital (CGMH), in Taiwan. CGMH is a 10,000-bed medical centre with 7 branches across Taiwan. Our study sample comprises emergency physicians from all CGMH branches. Ethical approval for this study was obtained from the Chang Gung Memorial Hospital Institutional Review Board (104-9298B).

### Characteristics of study participants

We recruited participants from emergency department across our hospital branches, using both purposeful and snowball sampling methods. Our inclusion criteria were that participants had to be currently employed as emergency physicians at one of the branches as either a resident or attending physician. All participants gave their informed consent before taking part in the study and were compensated for their time.

We collected data from 150 emergency physicians: 56 residents and 94 attending physicians. The sample had 128 males and 22 females with a mean age of 36.62 ± 7.48 years. 132 participants held a bachelors’ degree, 9 held a masters’ degree and 9 held PhDs. The average work experience was 2.23 years ± 1.24 and 10.54 years ± 6.58 for residents and attending physicians respectively.

### Procedure

The development of the Emergency Physicians’ Professional Identities Value Scale (EPPIVS) involved qualitative and quantitative approaches. Following Artino Jr, La Rochelle [23] guideline for scale development, we developed and validated the EPPIVS in two stages [23]. In the first stage we looked at the conceptualization of professional identities both in the broader context of medicine and then in the emergency medical environment. Data synthesis was used as the basis for our model and to generate items for the scale. The second stage involved testing our theoretical model by assessing its validity and reliability.

### Stage 1: Scale’s construct conceptualization and items generation

A thematic analysis of the literature on physicians’ professional identities provided an overview of the facilitators and barriers of professional identities across specialties. These results became the framework for a subsequent semi-structured interview exploring how n = 25 emergency physicians conceptualised their professional identities. The interviews yielded insights into physicians’ shared beliefs, extrinsic and intrinsic values about work culture, norms and professional identities [24]. We then ascertained the relative importance of these beliefs and values among a different sample of n = 33 emergency physicians through a Q-methodology study. Here, participants were required to rank value statements derived from our literature review and interviews on a Q sort grid according to their relative importance in shaping how they define themselves as emergency physicians. Using a correlation matrix and varimax rotation method of principal component analysis, the results revealed that, despite having collective beliefs and values, emergency physicians’ weighting of values that shape their view of what it means to be an emergency physician could be clustered into four varied viewpoints. These viewpoints were labelled (1) Skill acquisition, capabilities and practical wisdom, (2) Coping ability and resilience, (3) Professional recognition and self-esteem, as well as (4) Well-being and quality of life [2]. Based on these findings, we adopted these four factors as the latent variables underpinning physicians’ professional identities for our scale.

We generated the scale’s items from our Q-sort, literature review, semi-structured interviews results and by adapting items from existing scales. Thus, using existing professional identity scales in literature, we adapted the following items: “my work makes me feel satisfied” [25] and “being a nurse makes me happy” [26] to “gaining pleasure from professional work”; and “my profession should be the sole custodian of its skills, knowledge, and practices” [27] to “using clinical skills unfamiliar to general physicians”. Following our Q-sort findings, our research team held several discussions over 54 initially generated items’ wording, phrasing and back-to back translation between English and Chinese. As a result of these meetings, items were either modified or deleted until 40 items were left. These formed the preliminary version of our scale (see, Table 1, for the description of initial subscales and items).

### Emergency Physician Professional Identities Value Scale (EPPIVS) preliminary version

The 40-item preliminary version of EPPIVS was designed to measure physicians’ multidimensional professional identities through value evaluation. Phrased to reflect the core beliefs and values held by emergency physicians about key aspects of being an emergency physician [2], the scale had four subscales: Skill acquisition, capabilities and practical wisdom (12 items), Coping ability and resilience (8 items), Professional recognition and self-esteem (10 items), and Well-being and quality of life (7 items). The scale had three additional general items assessing physicians’ overall sense of belongingness and fit. A 7-point Likert scale ranging from 1 “not at all the same” to 7 “pretty much the same” is used to indicate the extent to which physicians’ personal values about their profession is similar to the scale’s statements.

### Stage 2: assessing scale’s psychometric properties

#### Content validity

To conduct content validity, we employed purposeful sampling method and assembled a panel of five physician experts from emergency medicine, neonatology, and gerontology specialties. The panel of experts worked independently to quantitatively rate and comment on the scale’s layout, item’s clarity, word usage, comprehensiveness, and relevancy to measurement constructs. Experts judged whether each item was 1-poor, 2-requires revision, 3-requires minor revision, 4-excellent and provided revision suggestions for items rated 2- requires revision and 3- requires minor revision. Our findings indicated that our scale was valid. Following iterative team discussions on the experts’ suggestions, 10 items were modified. For instance, the item “managing a wide range of *various medical situations*” was modified to “managing a wide range of *medical conditions*” based on experts’ suggestions.

### Construct validity and reliability

Based on our qualitative and Q-sort results, we hypothesised that the EPPIVS comprised four factors: Skill acquisition, capabilities, and practical wisdom; Coping ability and resilience; Professional recognition and self-esteem; and Well-being and quality of life. We also assumed that the four latent variables of our construct are inter-correlated with each other.

### Data analysis

Data were analysed using IBM’s SPSS 20 and IBM SPSS Amos 26 software. We first computed the descriptive statistics for the demographic variables and scale’s items. We checked the scale’s items normality by computing standard deviation, skewness, and kurtosis. We also checked for multivariate normality using Mardia’s normalized estimate of multivariate kurtosis and Mahalanobis distance. Given that our hypothesised model was based on our preliminary findings, we tested the construct validity using Confirmatory Factor Analysis (CFA) with maximum likelihood to confirm the relationship between the items and hypothesised factors (subscales).

To ascertain whether the model had reasonable model fit, we used a Chi-Squared test, normed chi square, Goodness-of-Fit Index (GFI), Comparative Fit Index (CFI), and Root Mean Square Error of Approximation (RMSEA). Chi-Squared test χ2, p > .05, with small χ2 value indicates a good fit [28] and GFI >. 80 indicates a goodness of fit [29]. A cut- off point of ≥ 0.90 is used for CFI and ≤ 0.08 is used for the RMSEA to indicate an acceptable model fit [30]. Iterative evaluation and adjustment of the hypothesised model was carried out following modification indices (MIs). To estimate internal consistency for items in each factor and the whole scale, we employed Cronbach’s α coefficient, composite, and McDonald’s Omega reliabilities. We used Alpha values α > 0.6 [31], CR values ≥ 0.7 [32] and McDonald’s Omega ≥ 0.7 [33] to indicate acceptable internal consistency. In addition to statistical criteria for item selection, criteria were employed to ensure that valid items are selected to reflect the constructs of interest. These include criteria related to how items are phrased to ensure accurate completion and suitable valuation, as well as the integrity of items grouping in terms of range of domains. This process helped to minimize response bias problems at the item selection stage.

## Results

### Confirmatory factor analysis

To assess items’ normality, skewness < 2, kurtosis < 3, critical ratios < 5.0 were set as cut-off points [31]. Our results show that the critical value which reflects Mardia’s normalized estimate of multivariate kurtosis was 21.87. This is greater than the 1.96, or 5.00 thus indicative of non-normality. The next step was to assess the multivariate normality and detect outliers using evaluating the Mahalanobis distance and its corresponding p values. A few cases indicated a low p value at the p < .01 level reflecting the presence of outliers. According to Keselman, Othman [34] psychological data rarely meets the normality assumptions, making the assessment of normality an important but still questionable practice in psychology. Further analysis of the outliers using box, scatter and Q-Q plots revealed that they were not extreme. Given our stringent p value cut-off point p < .01, sample size, sampling method, we decided to leave most outliers to maintain an unbiased view of the final scale. Since the multivariate normality assumption was violated, we proceeded to address this issue using a bootstrapping technique [35], setting the number of bootstrap samples to 100 which allowed us to control for normality. Testing the null hypothesis that the model is correct, Bollen-Stine bootstrap indicated that p = .010. Thus, we fail to reject the null hypothesis. The fit results were satisfactory. The final bootstrapped model has a Normed Chi square value = 2.374, a CFI = 0.862 and RMSEA = 0.096.

In general, all item’s responses were indicative of normally distributed responses except for item SAPW4, CAR3, and PRSE10 (see Table 1). These were subsequently eliminated in the construct validation process. We employed maximum likelihood estimation to test our hypothesised four-factor model with 37 items, excluding the three general items of the scale (see Table 1). A measurement model was developed for each domain first to test that each item belonged to the hypothesised latent factor. Following MIs, theoretical and methodological basis, we iteratively revised the items of each factor until the model reached statistical fit (See Table 1 for each domains’ items factor loadings before and after modification).

**Table 1:** Initial 4 subscales in the Emergency Physicians Professional Identities Value Scale with their respective items’ before and after modification factor loadings

Items Per Subscale	Factor Loading Before Modification	Factor Loading After Modification
**Skills Acquisition, Capabilities and Practical Wisdom (SAPW) (12 items)**		
Includes items reflecting physicians’ values on skills acquisition, competence and ability to handle the most challenging, complex and uncertain aspects of a physicians’ job		
1. Managing challenging patients effectively	0.721	0.737
2. Handling new work challenges	0.559	
3. Swift decision making around patients’ discharge	0.697	0.729
4. Solving clinical problems quickly	0.83	
5. Efficient multitasking	0.732	0.738
6. Teamwork to facilitate efficiency of everyday clinical work	0.444	
7. Utilising clinical skills that physicians in other specialties are unfamiliar with	0.56	0.557
8. Swift action for stabilising patients	0.863	
9. Diagnosing medical conditions quickly	0.911	0.885
10. Reflecting on work experiences for clinical skills improvement	0.613	
11. Maintain standard of care, despite medical disputes	0.587	0.598
12. Managing a wide range of medical conditions	0.692	0.67
**Coping Ability and Resilience (CAR) (8 items)**		
Includes items that reflect physicians’ values on their coping abilities both at work and outside work, ability to adapt to sudden change, to manage workload, handle pressure and remain calm.		
1. Good leadership to facilitate workforce stability	0.499	
2. Swift recovery from upset at work	0.687	0.688
3. Prioritising patients for care	0.544	
4. Staying positive when working under pressure	0.802	0.857
5. Having the ability to trust colleagues	0.655	0.647
6. Effective management of violence in the clinical setting	0.639	
7. Remaining calm when managing sudden events at work	0.689	0.642
8. Appropriate remuneration for professional services	0.449	
**Professional Recognition and Self-Esteem (PRSE) (10 items)**		
Includes items associated with physicians’ values around issues of recognition from the public, competence and possession of unique set of skills different from other physicians, and recognition of their contribution to the health care system		
1. Playing an important role in society	0.491	0.514
2. Gaining pleasure from professional work	0.607	0.656
3. Competence as a physician	0.523	
4. Contributing as a good leader in professional teams	0.613	0.613
5. Professional associations standing up for its’ members rights	0.547	
6. Organisational recognition around the value of physicians in my specialty	0.514	
7. Public recognition of my specialty as a profession	0.502	
8. Development of professional sub-specialties	0.595	0.659
9. Staying positive when facing patients’ complaints	0.737	0.653
10. Autonomy in decision making around patient care	0.57	
**Well-Being and Quality of Life (WBQL) (7 items)**		
Includes items related to physicians’ values on finding work-life balance, job satisfaction, finding purpose in their work and pursuit of personal well-being.		
1. Having a high level of emotional intelligence for workplace effectiveness	0.615	
2. Utilising personal values and beliefs to sustain professional work	0.749	0.646
3. Effective communication with patients	0.681	
4. Having a manageable workload	0.541	0.479
5. Happiness in one’s personal life	0.801	0.939
6. Engaging in leisure activities outside of work	0.489	
7. A work-life balance	0.689	0.677
**General (3 Items)**		
Includes items related to the physicians’ personal assessment of an overall sense of belongingness and fit		
1. Taking pride in one’s work		
2. I feel that I belong to the emergency medicine profession		
3. I feel that I fit my professional role as an emergency physician		

Skills acquisition and practical wisdom factor’s hypothesised structure of initial 12 items poorly fitted our data. One item SAPW4 was eliminated from the factor for lack of normality. Following MIs we omitted 4 items one at a time and our final factor structure with 7 items was a good fit (Model fit: χ2 = 15.29; df = 14; p value = 0.358; Normed χ2 = 1.092; GFI = 0.971; CFI = 0.997; RMSEA = 0.025) for our data. The initially hypothesised 8 item model for ‘coping ability and resilience’ factor poorly fitted our data. One item CAR3 was not normally distributed and thus eliminated along with 3 other items based on MIs’ suggestions. The final model with four items indicated the best fit for our data (Model fit: χ2 = 0.880; df = 2; p value = 0.644; Normed χ2 = 0.440; GFI = 0.997; CFI = 1.000; RMSEA = 0.000).

For the ‘professional recognition and self-esteem’ factor, we hypothesised that 10 items would load into this factor. One item (PRSE10) was not normally distributed and was eliminated along with 4 other items based on MIs’ suggestions. The final factor with 5 items best fitted our data (Model fit: χ2 = 5.356; df = 5; p value = 0.374; Normed χ2 = 1.071; GFI = 0.986; CFI = 0.998; RMSEA = 0.022). The initial 7-item structure of ‘well-being and quality of life’ factor did not provide a good fit for our data. Following MIs, we eliminated 3 items and covaried item WBQL 2 and WBQL 4. The final 4 item model had the best fit for our data (Model fit: χ2 = 1.637; df = 1; p value = 0.201; Normed χ2 = 1.637; GFI = 0.995; CFI = 0.997; RMSEA = 0.065).

**Fig. 1:**
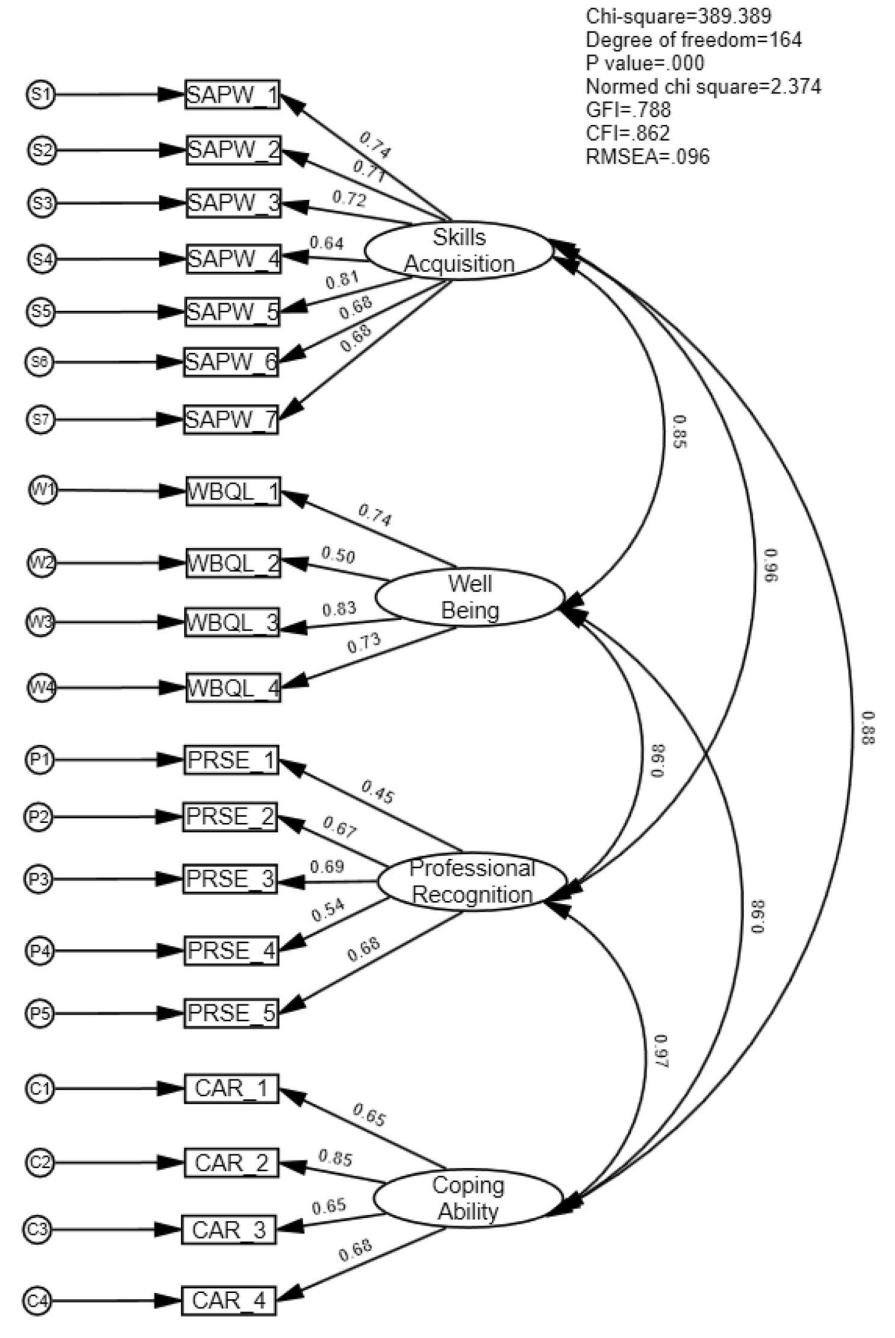
Bootstrapped model with error variance

A total of 20 items remained from the four factors forming the emergency physician professional identities’ measure. We assumed that these four factors were inter-correlated with each other and tested this assumption using CFA. Figure 1 shows the schematic representation of the final bootstrapped model and the goodness of fit statistics χ2 = 389.38, df = 164, p value = 0.000, Normed χ2 = 2.374; GFI = 0.788; CFI = 0.862; RMSEA = 0.096. Two items from the wellbeing subscale and professional recognition have weak factor loadings which may suggest weak convergent validity for this subscale.

.

The final EPPIVS version is a 20-item scale that measures professional identities comprising four dimensions of values that shape physicians’ professional identities (see Supplementary file). As hypothesised, inter-scale correlation analyses conducted among the EPPIVS subscales showed that they were significantly and positively correlated. The internal consistency reliability for the EPPIVS total scale (CR = 0.899; α = 0.938) and each domain scale (ranged from CR 0.757 to 0.851, Omega 0.759 to 0.868 and α 0.756 to 0.868) were all within a good range. The reliability coefficients for each subscale were as follows: Skills acquisition and practical wisdom (7 items) CR = 0.851; α = 0.868, Omega = 0.868, Coping ability and resilience (4 items) CR = 0.803; α = 0.800, Omega = 0.807, Professional recognition and self-esteem (5 items) CR = 0.757; α = 0.756, Omega = 0.759 and Wellbeing and quality of life (4 items) CR = 0.803; α = 0.794, Omega = 0.799. The results suggest that validity and reliability of our hypothesised four-factor structure in the EPPIVS are acceptable.

## Discussion

The lack of consensus in the conceptualisation and operationalisation of physicians’ professional identities as a construct has made its assessment hard. Therefore, our study set out to develop and validate a multidimensional measure for assessing the latent construct of emergency physicians’ professional identities. This scale’s development was based on our preliminary studies’ findings examining emergency physicians’ inter-subjective conceptualisation of professional identities[24]. The scale’s operationalisation was based on Q-methodology’s factor analysis results, showing a variation in how physicians prioritise their shared professional values [2]. Therefore, our scale provides a novel way to measure physicians’ professional identities from a multidimensional perspective drawing on a multi- method design for the conceptualisation and operationalisation of the construct.

The psychometric properties of the EPPIVS confirm the reliability and validity of using this scale to assess emergency physicians’ professional identities. In terms of construct validity, the current study tested the latent factor structure of EPPIVS using a CFA. The CFA results suggested that the four-factors model explained the data with significant substantial model fit. Emerging literature indicates that the cut-offs suggested by Hu and Bentler [30] are not the most appropriate [36, 37]. Rather cut-offs should be adjusted. The Dynamic Fit Index has been presented as a potential remedy for determining practical cut-off points for models [36]. We conducted a DFI analysis. However, we did not obtain any results. According to McNeish and Wolf [37], the unavailability of DFI cut offs may be due to poor differentiation between fit indices from correct and misspecified model distributions, which can occur in contexts with high sampling variability such as small samples. Therefore, in this study, we used Hu and Bentler’s cut-off points as indicators of misspecification. As per tradition they are still widely recognized as the standard cut-off points within academia despite their shortcomings. We understand that its history or origins is imbued in subjectivity, thus only relied on them as guidelines. The EPPIVS and its subscales were found to have good internal consistency (Cronbach’s and Omega > 0.75). With settings indicating stringent criteria for item inclusion and theoretical congruence with the findings from our previous Q method study [2], we can concluded that EPPIVS is a feasible measure of physicians’ professional identities.

Values held by members of a profession give us a unified perspective of the important professional norms and practices, thus tell us what shapes the professional identities of the members [9]. Our findings suggest the validity of measuring professional identities as a multidimensional construct with four latent factors based on diverse values rather than a unidimensional construct indicating high or low identification with the physicians’ profession. Based on the assumption that our identities are complex and multifaceted [38], we argue that a multidimensional scale offers a wider and in depth understanding of the construct. Our scale is grounded in the values related to professional practice, goals and standards that shape physicians’ perceptions about their self-image and identities within their profession.

Our scale is a valuable addition to the existing literature on value-based scales for healthcare professionals. For example, our Coping Ability and Resilience subscale shares similarities with the Medical Professionals Resilience Scale (MeRS), which measures medical professionals’ resilience when facing adversity at work [39]. Although the development processes of the two scales differ, the final items in our scale indicate similarities with the dimensions of MeRs. The four domains of the MeRS - control, resourceful, involvement, and growth - correspond to our four final items: remaining calm when managing sudden events at work, having the ability to trust colleagues, staying positive when working under pressure, and swift recovery from upset at work, respectively.

Additionally, our subscales share similarities with the Physician Values in Practice Scale (PVIPS) [40].Our coping and resilience subscale and wellbeing and quality of life subscale emphasize the value of being able to cope with the demands of one’s work, resembling the essence of the lifestyle dimension of the PVIPS. The prestige subscale of PVIPS shares similarities with our professional recognition and self-esteem subscale, both focus on physicians’ values of having outsiders perceive their specialty as having status and high standing. However, there is no overlap between the autonomy, management, scholarly pursuits, service PVIPS subscales with our own professional identity scale. Another value-based scale found in the literature is the Health-care Practitioner Values Scale (HPVS), which assesses the importance of 11 dimensions in guiding health professionals in their practice [15].

Overall, our conceptualization of values aligns with other value-based scales found in the literature, informed by the core values that are key tenants of the theory of work adjustment [41] and Schwartz’s values model [42]. Nevertheless, our development process involving interview studies and Q-methodology was crucial to highlighting the nuanced perspectives of emergency physicians on values that shape their professional identities and to refining the dimensions of values. This resulted in partial overlap between our identity scale and previous value-based scales.

When an individual enters a profession, they become a part of a community that shares a set of values and beliefs that guide their work. These values are measured by the by the generic value -based scales such as HPVS, and PVIPS. However, the process of internalization which is characteristic of identity development ensures that these shared values and beliefs become a part of the individual’s professional identity and shape their sense of purpose and self as a professional. Our newly developed identity scale measures these latter forms of values. This is what is unique and important about our scale.

From a values perspective, physicians hold all the values represented by the scale’s items. However, the perceived importance of some values varies. The EPPIVS measures the variation in the conceptualisation of beliefs and values underlying physicians’ profession, hence offers insight into physicians’ professional identities by highlighting different aspects of the construct. Additionally, by capturing these variations, the latent factors of our scale reflect the intersection of multiple identities and the constant negotiation between personal and professional values that occurs over the course of physicians’ careers as they develop their professional identities [2, 43]. Moreover, we propose that the multidimensional approach to identity assessment of the 20-item EPPIVS is in line with Van Knippenberg [44] levels of socialisation of newcomers into a community of practice through adoption of attitudes, behaviours, acquisition of core skills and competencies relevant to the community of practice [44, 45].

### Implications

The newly developed scale has the potential to reflect individual variation in the endorsement of shared values that underpin the practice of emergency medicine. Hence, it can facilitate the recruitment of residents through values-based career decision making, ensuring that work values align with the model of emergency care delivery. Determining the values that drew residents into the profession in the first place, is the first step towards providing tailored incentives that appeal to their individual values. This strategy can potentially contribute to the efforts of retaining physicians within the emergency medicine profession hence minimise loss of training resources incurred when physicians leave the profession [15, 16, 46]. Indeed, the scale has potential implications on the assessment of emergency physicians’ value internalisation patterns, hence identity development throughout their career. By providing an auxiliary conceptualisation of physicians’ professional identities as multifaceted and dynamic, our scale provides a way to capture not just snap shots of physicians’ professional identities but also to monitor the long-term changes in their professional identities. Such an evaluation could help program directors to monitor the legitimacy of the construction and the development of professional identities[47]. Furthermore, research promotes professional identity development of physicians as an important aspect for physicians’ continuous professional development [12, 38]. The scale’s four latent factors, wellbeing and quality of life, skills acquisition and practical wisdom, coping ability and resilience and professional recognition and self-esteem have potential implications in the development and evaluation of initiatives to support emergency physicians’ navigation through different challenges and stages [1].

### Limitations

Our study has several limitations. For example, the scale’s content was developed according to data from open-ended interviews using Q-sort, and the richness of the scale’s contents was judged by five physicians from different specialties. Through this approach, the content validity of the EPPIVS was ensured by item selection. However, only 5 physicians were interviewed, and this small sample size may not be sufficient to fully capture the complicated response process of physicians across different specialties. Therefore, cognitive debriefing regarding the item wording and meaning is necessary to ensure the response process validity of the EPPIVS in future studies. In addition, this study could have benefited from having a heterogeneous sample from wider communities of practice across different hospitals. The homogeneous institution culture of our sample may not provide an exhaustive list of physicians’ values and professional identities. Another limitation of the study is the gender gap that currently exists in the Taiwanese medical setting. The gender gap is even worse when it comes to the emergency specialty, wherein female physicians account for around 10%. As a result, our study had only 22 female participants.

As is widely known, validating, and refining a scale is an ongoing process, not a task that can be completed within a single cycle. Hence future studies could benefit from using a validation framework to guide the research process. Using this validation framework will allow us to identify which sources of evidence for validity have been examined in the current EPPIVS version and suggest subsequent steps for seeking the missing sources. Currently, we have demonstrated evidence for the internal structure of the tool in context, which mainly falls under the scoring components. Finally, we are also aware of the limitations presented by our sample size. Further evaluation and validation of the scale’s psychometrics is required, e.g., responsiveness across emergency physicians’ samples, scale and subscales’ scoring accuracy, criterion validity, reproducibility and stability, as well as predictive validity through hypothesis testing. Plans for a future study that will assess the scale using a larger sample from a wider range of hospitals including both private and public are already underway. This will strengthen the scale’s further validation process.

## Conclusions

The newly developed 20-item Emergency Physicians’ Professional Identities Value Scale (EPPIVS) can be a valid and reliable tool to access what values underpin emergency physicians’ professional identities. In contrast to previously developed scales, this scale measures physicians’ professional identities from a broader perspective using four dimensions; skills acquisition, capabilities, and practical wisdom, coping ability and resilience, professional recognition and self-esteem and wellbeing and quality of life, based on theoretical and empirical findings. Our findings may have important implications in ascertaining the varying degrees of physicians’ values in how they articulate their sense of self, skillsets, goals, wellbeing, and roles within their profession. This study contributes to the quantitative research on physicians’ professional identities. Building on our current findings, we will use this scale to examine the relation between professional identities and the outcome indicators of coping with practice in health settings such as stress and burnout.

## Electronic supplementary material

Below is the link to the electronic supplementary material.

Supplementary Material 1

## Data Availability

All data generated or analysed during this study are included in this article and its supplementary information files. Additional data is available from the authors upon reasonable request.

## Abbreviations

CFA: Confirmatory Factor Analyses
EPPIVS: Emergency Physicians Professional Identities Value Scale
GFI: Goodness-of-Fit Index
CFI: Comparative Fit Index
RMSEA: Root Mean Square Error of Approximation
MIs: Modification indices
SAPW: Skill acquisition, capabilities and practical wisdom
CAR: Coping ability and resilience
PRSE: Professional recognition and self-esteem
WBQL: Well-being and quality of life
