# Supplementary material for: Conceptualisation and Development of a values-based scale of emergency physicians’ professional identities
Source: BMC Med Educ. 2023 Jun 2;23:400. doi: 10.1186/s12909-023-04376-0 (PMC10236731; doi:10.1186/s12909-023-04376-0)
Supplement: Supplementary file 1 — Supplementary Material 1 [file 12909_2023_4376_MOESM1_ESM.docx]

**Supplementary file**

**Validated 20-Item Emergency Physicians’ Professional Identities Value Scale (EPPIVS)**

Values are a way of expressing who we are and what is important to us. Each individual’s values are important because they provide meaning to their life and therefore different people value different things. What do you value most about your profession?

Below is a set of work-related value statements underpinning the emergency medicine profession. Please indicate on a scale of 1 (not at all the same) to 7 (pretty much the same) the extent to which these values are the same as your own professional values right now:

We are not interested in what you think is the perfect or ideal professional values, we are interested in knowing which values you hold irrespective of whether or not you have attained those values.

Please note, there are no right or wrong answers - just your own opinion. Do not think too hard about any of the items, your initial reaction is usually the best.

|  |  | Not all the same | |  | |  | |  | |  |  | Pretty much the same | | |
| --- | --- | --- | --- | --- | --- | --- | --- | --- | --- | --- | --- | --- | --- | --- |
| 1 | Swift decision making around patients’ discharge | 1 | 2 | | 3 | | 4 | | 5 | | 6 | | 7 |  |
| 2 | Utilizing clinical skills that physicians in other specialties are unfamiliar with | 1 | 2 | | 3 | | 4 | | 5 | | 6 | | 7 |  |
| 3 | Having the ability to trust colleagues | 1 | 2 | | 3 | | 4 | | 5 | | 6 | | 7 |  |
| 4 | Playing an important role in society | 1 | 2 | | 3 | | 4 | | 5 | | 6 | | 7 |  |
| 5 | Happiness in one’s personal life | 1 | 2 | | 3 | | 4 | | 5 | | 6 | | 7 |  |
| 6 | Utilizing personal values and beliefs to sustain professional work | 1 | 2 | | 3 | | 4 | | 5 | | 6 | | 7 |  |
| 7 | Contributing as a good leader in professional teams | 1 | 2 | | 3 | | 4 | | 5 | | 6 | | 7 |  |
| 8 | Gaining pleasure from professional work | 1 | 2 | | 3 | | 4 | | 5 | | 6 | | 7 |  |
| 9 | Maintain standard of care, despite medical disputes | 1 | 2 | | 3 | | 4 | | 5 | | 6 | | 7 |  |
| 10 | Having a high level of emotional intelligence for workplace effectiveness | 1 | 2 | | 3 | | 4 | | 5 | | 6 | | 7 |  |
| 11 | Development of professional sub-specialties | 1 | 2 | | 3 | | 4 | | 5 | | 6 | | 7 |  |
| 12 | Swift recovery from upset at work | 1 | 2 | | 3 | | 4 | | 5 | | 6 | | 7 |  |
| 13 | Remaining calm when managing sudden events at work | 1 | 2 | | 3 | | 4 | | 5 | | 6 | | 7 |  |
| 14 | Having a manageable workload | 1 | 2 | | 3 | | 4 | | 5 | | 6 | | 7 |  |
| 15 | Staying positive when facing patients’ complaints | 1 | 2 | | 3 | | 4 | | 5 | | 6 | | 7 |  |
| 16 | Efficient multitasking | 1 | 2 | | 3 | | 4 | | 5 | | 6 | | 7 |  |
| 17 | Staying positive when working under pressure | 1 | 2 | | 3 | | 4 | | 5 | | 6 | | 7 |  |
| 18 | Managing a wide range of medical conditions | 1 | 2 | | 3 | | 4 | | 5 | | 6 | | 7 |  |
| 19 | Diagnosing medical conditions quickly | 1 | 2 | | 3 | | 4 | | 5 | | 6 | | 7 |  |
| 20 | Managing challenging patients effectively | 1 | 2 | | 3 | | 4 | | 5 | | 6 | | 7 |  |

Subscale scores were calculated by summing up scores for items belonging to each subscale, with a higher score indicating higher degree of professional identity.

**Subscale 1: Skills Acquisition, Capabilities and Practical Wisdom:** 1, 2, 9, 16, 18, 19, 20

**Subscale 2: Coping Ability and Resilience:** 3, 12, 13, 17

**Subscale 3: Professional Recognition and Self-Esteem:** 4, 7, 8, 11, 15

**Subscale 4: Well-Being and Quality of Life:** 5, 6, 10, 14

Please email us at [changyuche@gmail.com](mailto:changyuche@gmail.com) to get a copy of the Mandarin version of Physicians Professional Identity Value Scale.
